# The Role of Thrombospondins in Osteoarthritis: from Molecular Mechanisms to Therapeutic Potential

**DOI:** 10.7150/ijbs.103343

**Published:** 2025-03-03

**Authors:** Yirixiati Aihaiti, Hui Yu, Peng Xu

**Affiliations:** 1 Department of Joint Surgery, Xi'an Jiaotong University Affiliated HongHui Hospital, Xi'an, China.; 2 Key Laboratory of Pathogenesis and Precision Treatment of Arthritis, Xi'an, ShaanXi province, China.

**Keywords:** osteoarthritis, thrombospondin, extracellular matrix

## Abstract

Osteoarthritis (OA) is a prevalent chronic degenerative joint disorder characterized by cartilage degeneration, joint inflammation, and pain. The pathogenesis of OA still remains unclear. Among the various factors contributing to OA, the role of extracellular matrix (ECM) proteins, particularly thrombospondins (TSPs), has garnered significant attention. TSPs, a family of multifunctional extracellular matrix glycoproteins, are known to participate in numerous physiological and pathological processes, including cell adhesion, migration, differentiation, angiogenesis, and synaptogenesis through cell-cell and cell-matrix interactions. In this review, we provide a summary of the current understanding of TSP proteins in the pathogenesis of OA, including their effects on cartilage homeostasis, synovial inflammation, and subchondral bone remodeling and arthritic pain. We also review the evidence supporting the potential of TSP proteins as diagnostic biomarkers and therapeutic targets, with a focus on recent advances in cartilage regeneration, gene delivery therapy and pain management. Considering the multifaceted roles of TSP proteins in maintaining articular homeostasis, TSP proteins emerge as promising therapeutic targets for OA.

## 1. Introduction

Osteoarthritis (OA) is one of the most prevalent degenerative diseases among the elderly, characterized by progressive cartilage loss, osteophyte formation, chronic synovitis, and recognized as a leading cause of musculoskeletal disability worldwide. OA primarily affects weight-bearing joints, such as the knee and hip, presenting with clinical manifestations including gradually increasing joint pain, swelling, stiffness, and reduced mobility 1. In advanced stages, it can lead to joints deformity and disability. The etiology of OA remains unclear; however, genetic factors, anatomical abnormalities, sex, obesity, and trauma have been identified as risk factors 2. The global aging trends observed today have led to a rapid increase in the incidence of OA, affecting approximately 300 million individuals globally, with a higher prevalence in females 3. The current approach to OA treatment primarily aims to alleviate joint pain, improve joint function, slow disease progression, correct joint deformities, and consider joint replacement surgery as a last resort for end-stage disease due to the limited availability of disease-modifying osteoarthritis drugs (DMOADs) 4. Therefore, understanding the pathogenesis of OA is crucial for identifying diagnostic biomarkers and therapeutic targets at various stages of the disease, which can help arrest disease progression, improve patients' quality of life, and reduce strain on healthcare systems.

**Figure 1:**
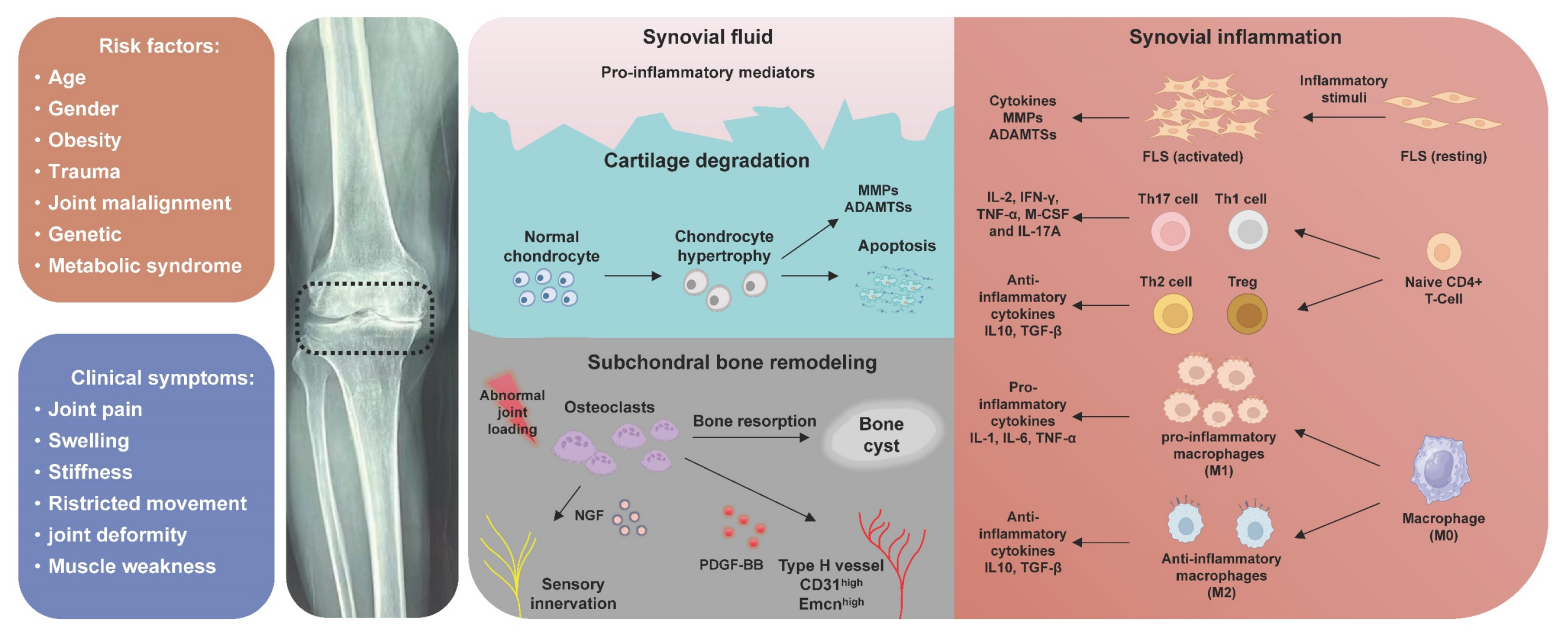
Pathological alterations of OA. The pathogenesis of OA involves the degeneration of cartilage, inflammation of the synovium, subchondral bone formation.

In recent decades, there has been a paradigm shift in the understanding of OA pathogenesis, transitioning from perceiving it as a mere 'wear and tear' effect on weight-bearing joint cartilage to recognizing it as a chronic disorder that impacts the entire joint. This includes cartilage degeneration, structural changes in subchondral bone, formation of osteophytes, synovial inflammation, as well as tendon and ligament degeneration (Figure 1). Aberrant extracellular matrix (ECM) remodeling is closely related to the pathogenesis of OA 5. ECM is an intricate acellular, three-dimensional structural network that is ubiquitously present in almost all tissue 6. The role of the ECM extends beyond providing biomechanical support to cells, tissues, and organs; it also maintains homeostasis of microenvironment through regulating numerous biological processes, including cellular adhesion, migration, differentiation, and growth 7. Thrombospondin (TSP) is a family of extracellular oligomeric glycoproteins consists of five distinct conserved members (TSP-1,2,3,4 and 5) 8. TSPs serve as not only structural proteins, but also contribute to tissue repair, angiogenesis, synaptogenesis, and tissue inflammation through mediating cell-ECM interactions 9. Despite being discovered over 40 years ago, specific roles of TSPs in the musculoskeletal system remain incompletely understood, with ongoing discoveries shedding new light. Therefore, a comprehensive review is required to clarify the physiological functions and molecular mechanisms of TSPs in the pathogenesis of OA. This review provides a thorough discussion regarding the involvement of TSPs in the pathological alterations during OA, emphasizing their potential as diagnostic biomarkers and therapeutic targets for OA. We also highlight the challenges and opportunities involved in translating these findings into clinical practice.

## 2. Structure and biological functions of thrombospondins

**Figure 2:**
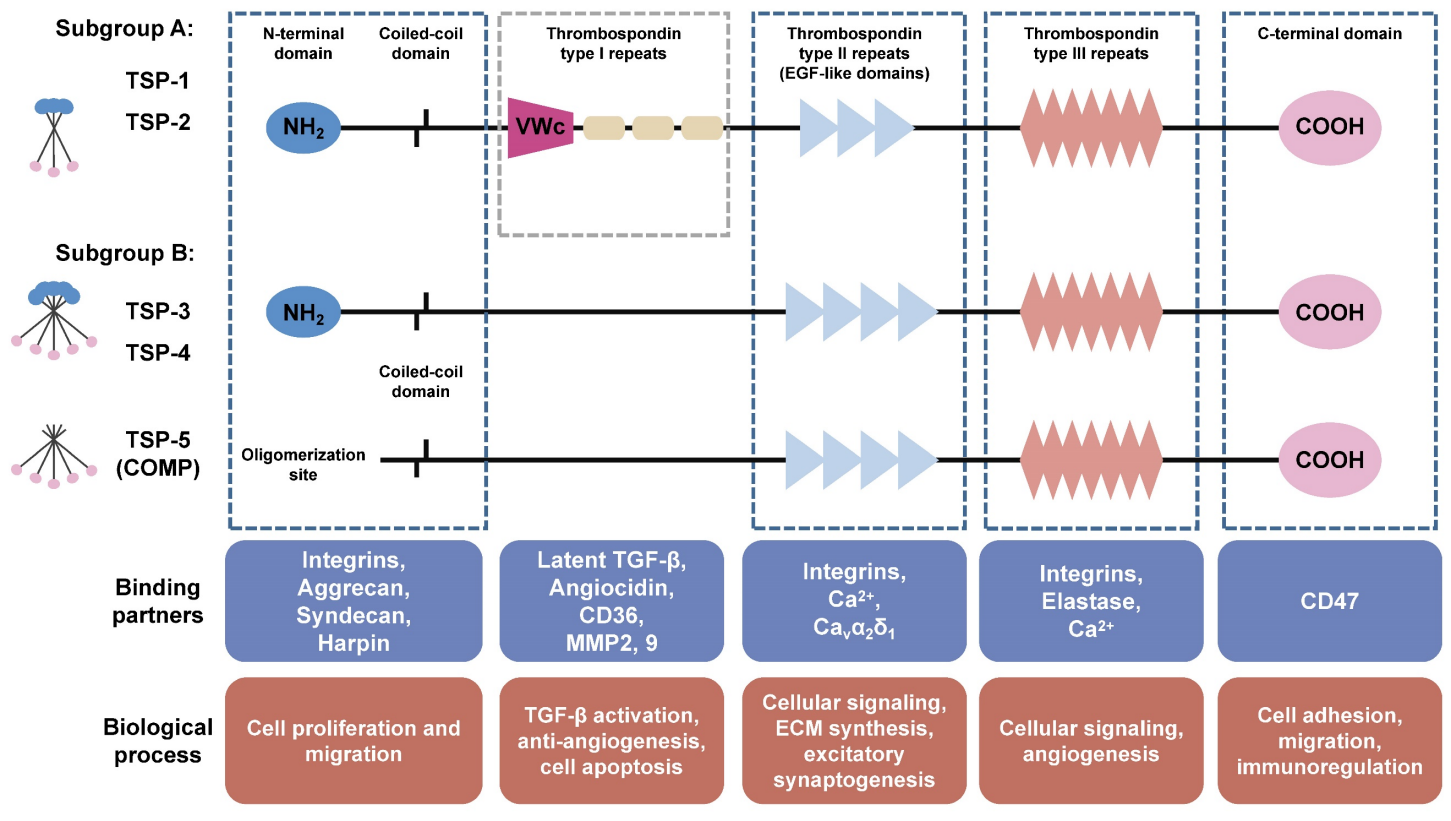
Structure and biological functions of TSPs. TSP family consists of five members (TSP-1, -2, -3, -4, and -5) which are classified into two subgroups. Subgroup A (trimer) comprises TSP-1 and TSP-2, while subgroup B (pentamer) consists of TSP-3, TSP-4, and TSP-5. The TSP subunits possesses a conserved feature of C-terminal domain contains CD47 binding site. TSP type III repeats are involved in angiogenesis through binding with Ca^2+^, integrins, and elastase. Type II epidermal growth factor-like (EGF-like) repeats are employed for regulation of ECM synthesis, migration, and excitatory synaptogenesis through binding with integrins, Ca^2+^, and Ca_v_α_2_δ_1_. The Von Willebrand factor type C (vWC) domain and type I repeats only exist in subgroup A and exhibit antiangiogenic activity by binding to CD36, and are implicated in binding and activation of TGF-β family. The N-terminal domains are much more varied in structure and sequence. TSP-5 lacks a typical amino-terminal domain. The coiled-coil oligomerization domain is responsible for homooligomers formation.

Structurally, the TSP family is classified into two subgroups 10. Subgroup A consists of TSP-1 and TSP-2, both possessing a trimeric structure and demonstrating similar functional properties. On the other hand, subgroup B consists of TSP-3, TSP-4, and TSP-5, which possess a pentameric structure (Figure 2). The TSP subunits possess a conserved feature of a C-terminal domain that contains tandem calcium-binding TSP type Ⅲ repeats, three (TSP-1 and TSP-2) to four (TSP-3, TSP-4 and TSP-5) type Ⅱ epidermal growth factor-like (EGF-like) repeats, as well as a carboxy-terminal domain structurally homologous to the L-type lectin domain. The C-terminal domain represents the distinctive hallmark of the TSP family 11, displaying a robust binding affinity to both collagenous and non-collagenous extracellular matrix proteins, thereby serving as a fundamental scaffold in the assembly of the collagen network 12. The N-terminal halves of TSP are much more varied in domain structure and sequence. The amino-terminal domains of subgroup A are identical and consist of an oligomerization domain, a von Willebrand factor type C (VWc) domain along with three thrombospondin-type Ⅰ repeats (TSRs), the subgroup B lacks both vWC domains and TSRs 8. Additionally, TSP-5 lacks a typical amino-terminal domain 13. Due to the multiple structural domains, TSP proteins exhibit various functions through binding with different cell surface receptors and ECM proteins. The VWc domains and TSRs in TSP-1 and TSP-2 exert antiangiogenic activity by binding to CD36 (a transmembrane glycoprotein) 14. In addition, TSRs are necessary for binding and activation of transforming growth factor (TGF)-β family. EGF-like domains are employed for regulation of cell adhesion and migration through binding of integrins and Ca^2+^. The C-terminal domain of TSP harbors a CD47 binding site, which consequently inhibits endothelial nitric oxide synthase (eNOS) activation and angiogenesis 8. TSP proteins are widely distributed in various tissues and organs including bone, cartilage, tendon, ligament, smooth muscle and synovial tissue, and exhibit a specific spatiotemporal distribution during the embryo development. Findings from knockout mice have revealed the specific physiological roles of TSP proteins in skeleton and cartilage development (Table 1) (extensive review for the modulation of TSPs in skeleton development, please refer to the reviews authored by Kurt D. Hankenson 15; 16). The expression levels of TSP proteins are low in normal tissues, but significantly increases following tissue injury, indicating the involvement of TSPs in the inflammatory response and the subsequent tissue remodeling including fracture healing, cartilage regeneration, and wound healing 17. Numerous studies have demonstrated the involvement of TSPs in the pathological progression of cardiovascular diseases, tumor genesis, metastasis and therapies response 17; 18. An in-depth exploration of the precise mechanisms through which TSPs participate in OA progression is crucial for the development of novel therapeutic strategies.

## 3. The role of Thrombospondins in the pathogenesis of OA

### 3.1. Thrombospondins in cartilage homeostasis and degeneration

The degeneration of cartilage is widely acknowledged as the hallmark feature of OA. The articular cartilage is composed of chondrocytes that are embedded within a highly organized ECM consisting of collagen fibers, proteoglycans, glycoproteins, and interstitial water 28. Type Ⅱ collagen serves as the primary structural component of cartilage, forming a highly crosslinked fibrous network that imparts both support and tensile strength to the tissue. This robust framework is further reinforced and stabilized by the presence of minor collagens, such as types Ⅸ and Ⅺ, which integrate into the network to enhance its functional integrity 29. The major proteoglycan aggrecan imparts elasticity and resistance to compression for cartilage through the osmotic pressure and negative charge of its glycosaminoglycan side chains (chondroitin sulfate and keratin sulfate) 30. Apart from these structural proteins, the complex collagenous network also contains non-structural glycoproteins, such as perlecan, decorin and fibronectin 30; 31; 32, primarily responsible for binding to collagen and proteoglycans, thereby enhancing the stability of the collagen network and facilitating the chondrogenesis through cell-cell and cell-matrix interactions. The TSP family is also classified as a group of nonstructural glycoproteins within articular cartilage and has been investigated as crucial factors in both the development and degradation of cartilage. The expression and distribution patterns of TSP proteins exhibit variations throughout the process of chondrogenesis, suggesting their distinct and specific roles in the development of cartilage, as well as important biomarkers for chondrocyte differentiation. Farrell *et al.* 33 investigated the distribution pattern of TSP-5 and TSP-4 during growth plate maturation in mice, revealing that TSP-5 predominantly localized around columnar chondrocytes within the proliferation zone and hypertrophic zone of naive cartilage. Starting from postnatal day 7, a widespread distribution of TSP-5 was observed across all layers of cartilage until endochondral ossification occurred. The pericellular localization of TSP-4 was consistently observed in the hypertrophic zone throughout the maturation process of the growth plate, exhibiting a distribution pattern similar to that of COL-X 33. The expression of TSP-2 was predominantly observed in the majority of proliferating chondrocytes within the femoral head and acetabulum on day 15 of embryonic mouse tissue. On day 18, TSP-2 was localized to the perichondria of developing bones such as basioccipital, scapula, and ulna 34.

TSP-1 protein was present in the pericellular and interterritorial cartilage matrix of the middle and upper deep zones 35. TSP-3 transcripts were observed in only differentiated chondrocytes 36. Functionally, TSP family participates in the maintenance of cartilage homeostasis through various signaling pathways. An in-vitro study using porcine chondrocytes demonstrated that TSP-5 and TSP-4 facilitate collagen and proteoglycan synthesis, thereby preserving the phenotype of chondrocytes through activating TGF-β-induced Erk1/2 signaling pathway, additionally, TSP-5 promotes chondrocyte migration and attachment 37. TSP-1 maintains cartilage homeostasis by promoting chondrocyte proliferation and extracellular matrix synthesis, inhibiting apoptosis, and enhancing autophagy in IL-1β-induced chondrocytes 38. Relevant to this discovery, collagen content was significantly decreased in hearts of TSP-1 null mice, leading to enhanced production of MMP-3 and MMP-9 under pressure overloading conditions, consequently, resulted in early cardiac hypertrophy and dilation 39. TSP-2 null mice display connective tissue abnormalities, including irregular collagen fibrillogenesis, which may affect the structure and function of cartilage 34. TSP proteins may also serve as diagnostic biomarkers for OA, as their expression exhibited significant disparities between OA patients and healthy individuals. The expression of TSP-5 was consistently observed throughout all layers of normal cartilage, whereas the expression of TSP-4 was minimal 33. Conversely, the expression of TSP-4 was significantly increased in OA cartilage, whereas the TSP-5 was notably degraded 37. The expression of TSP-4 is correlated with disease severity of OA cartilage 40. TSP-4 can be detected in the serum of both healthy individuals and OA patients. Notably, amounts of TSP-4 fragments are specifically increased in the serum of OA patients, suggesting its potential in serological marker for OA diagnosis. In mild to moderate OA cartilage, an increased number of TSP-1 expressing chondrocyte were observed in the superficial region. As the degradation of matrix in severe OA cartilage, a strong reduction of TSP-1 producing chondrocytes and increased number of CD36 (a classical TSP-1 receptor) positive chondrocyte were observed 35. Similarly, in a rat model of OA, the expression of TSP-1 gradually decreases in articular cartilage along with disease progression. Additionally, the serum levels of TSP-1 also exhibit a decrease starting from 60 days after ACLT surgery 38. Moreover, TSPs also show promising potential as therapeutic targets for OA. Intraarticular injection of adenoviral carrying TSP-1 cDNA effectively attenuated OA progression by down-regulating MMP-13 expression in cartilage and reducing microvessel density and macrophage infiltration in synovial tissue 41. Collectively, the TSPs serve as crucial components of ECM and play a significant role in maintaining cartilage homeostasis.

### 3.2. Thrombospondins in synovial inflammation

The majority of patients with OA commonly present symptoms of synovitis, the presence of which is associated with the progressive deterioration of OA joint 42. In normal joint cavity, synovial tissue typically consists of 1-3 layers of synoviocytes forming a protective covering over the inner lining surface of the joint capsule, participating in maintaining articular homeostasis. Resident synoviocytes primarily consist of macrophage-like synoviocytes (MLSs) and fibroblast-like synoviocytes (FLSs). The main functions of MLSs include phagocytosis of debris from cartilage and meniscus, as well as immunoregulation. FLSs possess the ability to secrete hyaluronic acid (HA)-enriched synovial fluid, providing nourishment to cartilage and facilitating joint lubrication 43. Histopathologically, OA synovium demonstrates mild-to-moderate inflammation characterized by synovial lining hyperplasia, pannus formation, and immune cells infiltration 44. The level of immune cells infiltration in the synovial tissue of patients with OA is comparatively lower than that seen in individuals with rheumatoid arthritis (RA), but higher than what is observed in healthy individuals 45. The application of single cell RNA (sc-RNA) sequencing has also unveiled the existence of diverse immune cells within OA synovial tissue 46; 47. The expression of TSPs in OA synovial tissues exhibit significant difference compared to those observed in normal synovial tissues, as evidenced by multiple researches (Table 2). Maerz *et al.* utilized single cell RNA sequencing to identify patterns of outgoing cellular communication of TSPs within the synovium of OA mice, suggesting that TSPs may serve as important signaling molecules in the pathogenesis of OA 48. In addition to preserving cartilage integrity, TSP-1 exerts anti-arthritic effect by inhibiting angiogenesis and macrophages infiltration through activating TGF-β production in OA synovial tissue 41. However, in a contrasting conclusion, Decana *et al.* observed an increase of TSP-1 protein expression in the inflammatory synovial tissue from rat model of RA, and TSP-1 protein expression were positively correlated with articular destruction severity. Interestingly, treatment with TSP-1-derived peptide not only inhibited TSP-1 expression but also decreased pannus formation, neovascularization, inflammatory cells infiltration and cartilage destruction in articular joint 49; 50. The plasma samples from RA patients also exhibited elevated levels of circulating TSP-1 protein 51. Similarly, the expression of TSP-2 was significantly upregulated in OA-FLSs, exhibiting a positive correlation with inflammation level in synovial tissue. Elevated TSP-2 expression in OA-FLSs promotes IL-6 production by activating PI3K/AKT/NF-κB signaling pathway. Targeted therapy with a neutralizing antibody against TSP-2 attenuates articular cartilage degradation and suppressed IL-6 production in OA mice 52. Despite being known as angiogenesis inhibitors (to be discussed in 3.4), both TSP-1 and TSP-2 appear to function as pro-inflammatory mediators that promote synovial hyperplasia, cartilage degeneration and immune cells infiltration in synovial tissues. MLSs are the main source of inflammatory cytokines in OA joints, as the pro-inflammatory macrophages (M1) accumulate more in OA synovial tissue, promoting cartilage degradation through secreting pro-inflammatory cytokines, such as IL-1β, IL-6, and TNF-α 53. Modulating the polarization of synovial macrophages presents a promising therapeutic approach for OA. Previous studies have demonstrated the regulatory roles of TSPs in modulating macrophage polarization, with distinct members exhibiting diverse functions. The expression of TSP-4 is significantly increased upon the stimulation of pro-inflammatory cytokines (LPS, IFNγ, and GM-CSF) in bone marrow-derived macrophages (BMDM), furthermore, deficiency of TSP-4 promotes the polarization of BMDM into anti-inflammatory phenotype (M2), as evidenced by increased expressions of Egr-2 and Arg-1 54. Targeted inhibition of TSP-4 in macrophages within the inflammatory site has the potential to attenuate inflammation and promote tissue regeneration by promoting M2 macrophages polarization. TSP-2 promotes M2 macrophages polarization and inhibits apoptosis in murine alveolar macrophage cell through activating PI3K/AKT signaling pathway 55. However, TSP-1 exhibits dual roles in macrophages, exerting both pro-inflammatory and anti-inflammatory functions, on the one hand, TSP-1 stimulates TNF-ɑ production in macrophages through activating Toll-like receptor. Macrophage-specific Tsp-1 deletion protects mice against non-alcoholic fatty liver disease through reducing liver inflammation and fibrosis 56; 57. On the other hand, TSP-1 also acts as an inhibitor of local inflammatory response while facilitating tissue repair through the promotion of M2 macrophage polarization 58. Further investigation is warranted to elucidate the specific roles of TSP proteins in OA synovial inflammation, thereby offering potential therapeutic targets for the treatment of OA.

### 3.3. Thrombospondins in uncoupled subchondral bone remodeling

**Figure 3:**
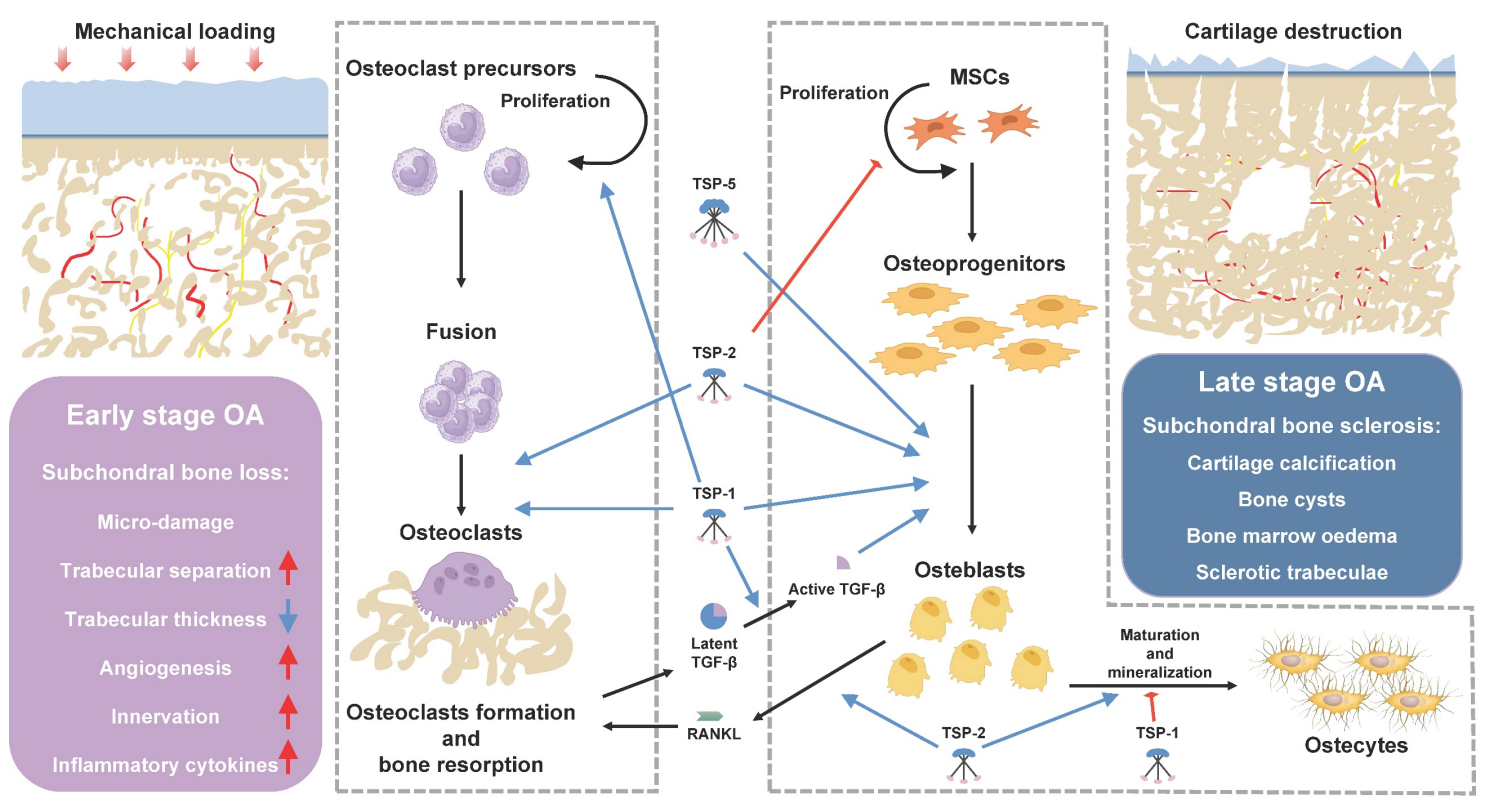
The role of TSPs in bone remodeling. In early-stage OA, subchondral bone is mainly characterized by osteoclasts-mediated bone resorption. Subchondral bone becomes porous with enlargement of trabecular gap and bone marrow cavity. Angiogenesis and sensory innervation were increased within subchondral bone. The TSPs play different roles in osteoclastogenesis. TSP-1 activates osteoclastogenesis through promoting proliferation and fusion of osteoclasts precursors, additionally, it acts as a major regulator of latent TGF-β activation released during bone resorption. TSP-2 facilitates the fusion of osteoclasts precursors and RANKL-dependent osteoclasts formation. In late-stage OA, subchondral bone is mainly characterized by osteoblast-mediated bone formation. Subchondral bone demonstrates increased bone density, bone volume, as well as decreased calcium to collagen ratio, bone mineralization, and mechanical stiffness. TSP-2 inhibits the proliferation and adipogenic differentiation of MSCs, and promotes osteogenic differentiation and bone deposition. TSP-1 and TSP-5 promote MSCs proliferation and osteogenic differentiation through activating TGF-β/smad signaling pathway.

Subchondral bone refers to the subchondral bone plate and subchondral bone trabecula distal to the tidemark of articular cartilage. The subchondral bone is the essential mechanical and nutritional support system of joints, maintaining the integrity and biological function of overlying cartilage 62. Under physiological conditions, subchondral bone remodeling is exquisitely regulated by osteoblasts-mediated bone formation and osteoclasts-mediated bone resorption 63. Distinct microstructural alterations occur in the subchondral bone at different stages of OA, even prior to the development of significant cartilage damage 64. In the early stage of OA, the number of osteoclasts markedly increases within subchondral bone, and the ratio of receptor activator of nuclear factor κ ligands (RANKL)/ osteoprotegerin (OPG) was elevated in osteocytes, thereby inducing the osteoclastogenesis and bone resorption 65; 66; 67. As a result, subchondral bone becomes porous with enlargement of trabecular gap and bone marrow cavity, decrease in subchondral bone plate thickness. Bone marrow edema, bone cyst and microfractures in subchondral bone can be detected through imaging examination 1; 68. Osteoclasts are multi-nucleated giant cells formed from the fusion of multiple monocytes/ macrophages, exhibiting positive staining for tartrate-resistant acid phosphatase (TRAP). Osteoclasts precursor cells differentiate into mature osteoclasts under the stimulation of macrophage colony-stimulating factor (M-CSF) and RANKL, with M-CSF promoting their proliferation and RANKL promoting their differentiation 69. The recruitment, differentiation, and activation of osteoclasts in early OA are primarily attributed to aberrant biomechanical and biochemical factors 70, however, the underlying mechanism remains unclear. TSP proteins have been assigned for multiple functions in osteoclastogenesis through binding with specific ligands (Figure 3). In a co-culture system with myeloma cells, immature dendritic cells transdifferentiate into TRAP-positive bone-resorbing multi-nucleated giant cells with significant upregulation of TSP-1 expression. Meanwhile, autocrine secretion of TSP-1 by osteoclasts precursors binds to CD36 and CD47, resulting in the inhibition of nitric oxide synthesis and promotion of monocyte fusion and osteoclastogenesis 71; 72; 73. Neutralizing antibody against TSP-1 addition led to significant inhibition of parathyroid hormone (PTH) induced hypercalcemia, osteoclasts formation and bone resorption both *in-vitro* and* in-vivo* 74. Consistently, TSP-1 knockout mice exhibited elevated bone mass and cortical bone size, accompanied by reduced bone resorption and osteoclasts formation, as well as increased expressions of inducible nitric oxide synthase (iNOS) 20. Lung cancer patients with high TSP-2 expression exhibited an increased susceptibility to bone metastasis 75. Lung cancer-secreted TSP-2 facilitates the RANKL-dependent osteoclasts formation in murine osteoclasts precursor RAW264.7 cells by activating NFATc1 and suppressing miR-486-3p expression, also modulating the RANKL/OPG ratio in osteoblasts. The inhibition of TSP-2 expression significantly impedes the bone metastasis of lung cancer cells *in-vivo* 76. Relatively, little is known about contributions of other TSP proteins in the process of osteoclastogenesis. The transient activation of osteoclasts leads to an increase in trabecular gap and bone marrow cavity, thereby promoting angiogenesis and innervation of the subchondral bone, concurrently stimulating osteoblastic bone formation through releasing transforming growth factor-β (TGF-β) from bone matrix 68; 77. Therefore, in the late stages of OA, subchondral bone is mainly characterized by osteoblasts-mediated bone formation, as evidenced by imaging examination revealing subchondral bone sclerosis and osteophytes formation 1. Subchondral bone in advanced OA patients demonstrates increased bone density, bone volume, and collagen content, as well as decreased calcium to collagen ratio, bone mineralization, and mechanical stiffness 78; 79; 80. TGF-β plays an important role in bone formation, through increasing osteoprogenitors proliferation and maturation, while inhibiting late stage osteoblast differentiation and bone matrix mineralization 81. TGF-β expression is significantly upregulated in the subchondral bone of both OA patients and mouse model of OA, leading to enhanced recruitment and osteogenic differentiation of mesenchymal stem cells (MSCs) within the subchondral bone 82. The regulation of TGF-β primarily occurs during the conversion of its latent precursor into the biologically active molecule. Specifically, the binding of the N-terminal latency-associated peptide (LAP) impedes TGF-β from engaging with its receptors, and it is imperative to disrupt this interaction for TGF-β signaling activation 83. TSP-1 is a major regulator of latent TGF-β activation, the KRFK sequence in TSP-1 type 1 repeats binds to a conserved sequence, LSKL, in LAP, disrupting LAP-mature domain interactions and activating TGF-β through exposing its receptor binding sequences (reviewed in 19). Aesculetin, a coumarin derivative, enhances osteogenic differentiation and bone matrix mineralization in the MC3T3-E1 cell line. Notably, aesculetin significantly accelerates the synthesis of TSP-1 and tenascin C in mature osteoblasts, facilitating their adhesion to preformed collagen matrix 84.

Therefore, the authors hypothesized that TSP-1 may participate in OA subchondral bone remodeling through activating TGF-β and promoting the osteogenic differentiation of MSCs, resulting in increased bone volume but decreased bone mineralization and mechanical stiffness in subchondral bone. By inhibiting the activity of TSP-1, it is possible to block the activation of TGF-β and potentially restore the uncoupling subchondral bone remodeling, thus presenting a potential therapeutic target for OA. Hankenson KD et. al demonstrated that MSCs from TSP-2-null mice exhibited increased proliferation and adipogenesis, decreased terminal osteoblastic differentiation, collagen fibrillogenesis and mineralization *in vitro*, suggesting that TSP-2 promotes osteoblasts differentiation and bone deposition *in vitro* 85; 86; 87; 88. Their *in vivo* findings demonstrated a significant upregulation of TSP-2 expression during the process of fracture healing. Additionally, TSP-2 deficient mice exhibited an increased formation of endocortical bone and cortical thickness due to an enhanced proliferation of osteoblasts progenitors. Moreover, TSP-2-null mice displayed augmented vascularization and a shift towards an intramembranous healing phenotype in ischemic fracture 23. They also observed that the mutation of TSP-2 provides protection against ovariectomy-induced bone loss through increasing osteoblastogenesis and inhibiting bone resorption 89. Bone morphogenetic proteins (BMP-2), also a member of TGF-β family, has been clinically applied for nonunion and lumbar body fusions 90. However, the application of BMP-2 is limited due to the requirement of supraphysiological doses 91. D.R. Haudenschild and colleagues revealed that TSP-5 also exhibits binding affinity towards TGF-β family, including TGF-β1 92 and BMP-2 93, thereby enhancing the osteogenesis through activation of TGF-β/smad signaling pathway, this binding interaction reaches its maximum potency under mildly acidic (pH 5.50-6.50) conditions with the presence of manganese 93. Comparison of the secretome from osteoblasts derived from sclerotic and non-sclerotic subchondral bone in OA patients revealed a significant reduction in the secretion of TSP-4 by osteoblasts from the sclerotic region 94. In contrast, Michael *et al.* reported a significant increase in TSP-4 mRNA expression during the osteoblastic differentiation process of primary murine osteoblasts 26. Additionally, Sofat *et al.* employed high throughput microarray analysis to investigate the genetic alterations in the subchondral bone marrow lesion (BML) from advanced OA, mild OA and normal individuals. Their findings revealed a striking increase of TSP-4 expression in BML regions of OA patients, accompanied by significant activation of pathways associated with angiogenesis (see in 3.4), pain sensitization (see in 3.5) 95. However, the specific roles of TSP-4 in regulation of osteoclasts and osteoblasts differentiation remain unclear. Collectively, these findings provide support for the distinct roles of TSPs in maintaining bone homeostasis. The aberrant expression of TSPs in the pathogenesis of OA may serve as a pivotal factor influencing subchondral bone remodeling.

### 3.4. Thrombospondins in angiogenesis

Angiogenesis consistently accompanies subchondral bone remodeling in the pathogenesis of OA. A distinct subtype of blood vessels, known as type H vessels, was identified in the trabecular bone adjacent to the growth plate and exhibited a notable expression of CD31 and endomucin (Emcn) 70; 96. In addition to oxygen supply, type H vessels are highly coupled with bone formation activities by attracting a large amount of osteoprogenitors around, regulating the osteoblasts differentiation 97. The balance between proangiogenic and antiangiogenic activities in subchondral bone is distributed during the pathogenesis of OA. In 60% of patients with OA, blood vessels breach the tidemark and infiltrate into avascular cartilage and meniscus, while vascular density within subchondral bone increases with disease progression and shows a positive correlation with histological severity score 98; 99. Targeted inhibition of angiogenesis in the subchondral bone represents a promising strategy for delaying the progression of OA. Numerous studies have elucidated the regulatory roles of TSPs in angiogenesis. Among the five members, TSP-1 and TSP-2 are renowned for their antiangiogenic effects, whereas the remaining three members exert proangiogenic effects. TSP-1, the first known endogenous anti-angiogenic protein, interacts with CD36 via TSRs and with CD47 via C-terminus, suppresses endothelial cell proliferation, migration, adhesion, and capillary-like structure formation, induces endothelial cell apoptosis through inhibiting NO/cGMP and vascular endothelial growth factor (VEGF) signaling pathway 8; 72; 100. TSP-2 inhibits angiogenesis by suppressing Notch signaling pathway 101. In contrast, Adognravi et. al reported that TSP-4 located within the lumen of growing vessels, and demonstrated that TSP-4 enhances endothelial cell proliferation, migration and adhesion through activating integrin α2/ TGF-β/Smad3 signaling pathway. TSP-4 deficiency resulted in impaired angiogenesis, delayed wound healing, and delayed postnatal vasculature development in mice 102; 103. In addition, TSP-4 overexpressing BMSCs increased the proliferation, migration, and capillary formation of human umbilical vein endothelial cells (HUVECs) through activating TGF‑β/Smad2/3 signaling pathway 104; 105. The specific role of TSP-5 on angiogenesis has not been fully elucidated yet. However, Chou *et al.* have developed a stable and soluble variant of Angiopoietin-1 (Ang1) named recombinant COMP-Ang1 by substituting the N-terminal region of Ang1 with the short coiled-coil domain of TSP-5. Comparing to the naive Ang1, this modified variant exhibits enhanced potency in promoting wound healing and bone defect healing through increasing angiogenesis, osteoblasts differentiation and bone formation 106; 107; 108; 109. Due to their distinctive antiangiogenic and proangiogenic properties, TSP proteins may serve as promising therapeutic targets for modulating subchondral bone angiogenesis in the progression of OA.

### 3.5. Thrombospondins in nerve sensitization

Pain is the primary reason patients with OA seek medical advice, as it limits joint function and reduces quality of life. For decades, the pain associated with OA has been attributed to nociceptive pain resulting from progressive joint degeneration, however, the clinical efficacy of pain management in OA patients remains suboptimal. Approximately 25% of the patients with OA reported experiencing pain characterized by neuropathic-like features, such as allodynia and hyperalgesia, suggesting the presence of central neural sensitization and additional mechanisms contributing to pain 110; 111; 112. Microarray analyses of the dorsal root ganglia (DRG) from experimental OA mice also revealed the existence of neuro-inflammation and immune response in OA-related pain 113; 114. The primary sources of nociceptive pain related to OA arise from the subchondral bone, synovium, meniscus, periarticular tendon, and ligaments, patients do not perceive cartilage degeneration due to its lack of innervation. Increased expressions of nociceptive neuron markers in the subchondral bone were observed from 1-week post-surgery in OA mice. The process of osteoclasts mediated bone resorption induces sensory innervation in the subchondral bone and increases hyper-excitability of DRG neurons by secreting Netrin-1 and nerve growth factor (NGF) 62; 115. All TSPs, both trimeric and pentameric isoforms, interact with the calcium channel alpha-2-delta-1 subunit (Ca_v_α_2_δ_1_) through their conserved EGF-like repeats, significantly increase excitatory synapse formation 116. Among all, TSP-4 has been extensively studied in synaptogenesis and is identified as a potential biomarker for pain assessment 117. The serum concentrations of TSP-4 protein were substantially elevated in patients with lumbar disc herniation or coronary artery disease during the acute painful phase, subsequent procedures such as intervertebral discectomy or percutaneous coronary intervention led to a decrease of TSP-4 protein concentrations in serum 117. Additionally, TSP-4, but not other TSPs expression is concurrent with the development of pain states and was positively correlated with VAS score 118. Mechanistically, peripheral nerve injury induces the expression of TSP-4 in spinal cord and DRG. David Luo et. al demonstrated that TSP-4 directly interacts with its receptor Ca_v_α_2_δ_1_ on the central terminals of sensory neurons, thereby promoting excitatory synaptogenesis and elevating sensory neurons excitability through decreasing high-voltage-activated (HVA) calcium current and increasing low-voltage-activated (LVA) calcium current 117; 119; 120; 121. During the process of nerve sensitization, TSP-1 plays a multifaceted role by not only promoting the sensitization process but also participating in pain resolution mechanisms. TSP-1 deficient adult mice exhibit reduced proliferation of neural progenitor cells and impaired neuronal differentiation, indicating that TSP-1 may positively regulate neuronal differentiation and synapse formation 122. Single-cell RNA sequencing result showed that TSP-1 expression was significantly increased in neutrophils and macrophages upon skin injury, and TSP-1 expression was positively correlated with the development of pain hypersensitivity. On the other hand, TSP-1 was shown to counteract prostaglandin E2 (PGE2)-induced sensitization of nociceptors, suggesting that TSP-1 is also implicated in the resolution of pain 123. Although the specific role of TSP in OA-related pain has not been reported, it is reasonable to hypothesize that TSP proteins may be involved in neuropathic pain state in OA by promoting excitatory synaptogenesis.

## 4. Therapeutic Potential of thrombospondins in OA

### 4.1. Cartilage regeneration

Due to its avascular nature, low cell density, limited nutrient supply and low proliferative activity, cartilage exhibits restricted intrinsic repair and regenerative capability following defects and degeneration. MSCs-based cartilage repair offers a promising therapeutic approach for the cartilage defects 124. However, the implanted seed cells failed to fully differentiate into mature hyaline cartilage; instead, they underwent ossification or fibrosis by activating the TGF-β/smad3 signaling pathway and vascular invasion. Moreover, the implanted seed cells exhibit deficient capacity in ECM synthesis, resulting in inferior compressive mechanical property and elasticity 125; 126. Thus, modifying seed cells is crucial to ensure their directional differentiation into chondrocytes, making it an important method for enhancing the success rate of cartilage repair. In addition to being a key glycoprotein within the cartilaginous ECM, TSP proteins also demonstrate the capacity to promote chondrogenic differentiation of MSCs, thereby facilitating effective cartilage regeneration (Table 3). Researchers from different groups employed lentivirus vector-mediated transfection of TSP-1 cDNA or recombinant TSP-1 in adipose-derived stem cells (ADSCs). Their investigation revealed that TSP-1 transfected ADSCs exhibited an increased anti-inflammatory property and chondrogenic differentiation, characterized by an increased content of collagen Ⅱ and glycosaminoglycans, as well as a decreased expression of collagen I, RUNX2, OCN, and OPN 59; 127. Similarly, TSP-2 enhances the chondrogenic differentiation of ADSCs and inhibits their hypertrophic maturation through the activation of PKCa, ERK, p38/MAPK and JAGGED1/NOTCH3 signaling pathways. Reversely, the depletion of TSP-2 expression leads to an increase in levels of hypertrophy-related genes (RUNX2 and MMP-13) and decrease in SOX9, Aggrecan and collagen II in the cartilage microspheres of MSCs 128; 129; 130; 131. Furthermore, TSP-5 has also been reported to facilitate cartilage regeneration. The up-regulation of Tsp-5 mRNA precedes Col2a1 by several days during chondrogenic differentiation of BMSCs. In a rabbit osteochondral defect model, the implantation of TSP-5-overexpressing MSCs loaded-biphasic scaffold significantly enhances hyaline cartilage formation and improves the mechanical properties of the newly formed cartilage 132; 133; 134; 135. Compared to other members, TSP-3 and TSP-4 are relatively less studied in chondrogenic differentiation. Wan-Ju Li et. al established reprogrammed MSCs (Re-MSCs) through overexpressing pluripotency factors in synovial fluid-derived MSCs from OA patients (Pa-MSCs). The analysis of chondrogenesis revealed that Re-MSCs demonstrated a higher capacity for differentiation into mature articular cartilage, with an elevated expression of TSP-4 potentially accounting for the enhanced chondrogenic differentiation ability of Re-MSCs 136. Collectively, TSP proteins exhibit promising potential in cartilage regeneration.

### 4.2. Pain management

Due to the lack of effective treatment options, analgesics remain the primary choice for managing OA symptoms so far. A stepped treatment approach including paracetamol, non-steroidal anti-inflammatory drugs (NSAIDs), steroids and opioids is employed based on the progression of the disease and severity of pain 139.

However, the side effects of these drugs are inevitable. It is crucial to recognize the molecular mechanisms responsible for initiating and sustaining the OA-related pain, with the aim of creating more potent therapeutic agents. Peripheral nerve injury induces up-regulation of TSP proteins in DRG that contribute to the development of neuropathic pain states 118, therefore, targeting TSPs could potentially serve as a therapeutic approach for pain management, including in OA patients. Intrathecal injection of TSP-4 antibodies, antisense oligodeoxynucleotides, or gabapentin reverses behavioral hypersensitivity and established allodynia in a rat model of spinal nerve ligation injury 118; 120. Gabapentin, an antiepileptic and analgesic drug, competitively binds to Ca_v_α_2_δ_1_ subunit, reduces its interaction with TSPs, which results in a reduction of calcium ions influx and inhibition of excitatory synapses formation (but not in already formed synapses) 116. Gabapentin has been reported to improve disease-related pain in animal models of arthritis. In adjuvant-induced arthritis (AIA) rat model, gabapentin treatment led to a significant improvement in the general condition of the rats, including a reduction in paw swelling and an increase in paw withdrawal mechanical threshold (PWMT), reducing the expression of FGF2 and FGFR1 in the DRG through the upregulation of miR-15a 140. In monosodium iodoacetate-induced arthritis (MIA) rat model, pregabalin significantly inhibited the neuronal responses to noxious electrical, mechanical, and thermal stimuli in OA rats, indicating its potential as an analgesic agent 139. However, the analgesic effects of gabapentinoids in experimental arthritis are state-dependent. They are more effective in the presence of central sensitization, with a more pronounced impact on neuropathic pain conditions, but do not influence baseline sensory neuron excitability or sensory thresholds in control animals 120; 141. The mechanisms underlying this effect, including whether it involves the inhibition of TSP-4-induced synaptogenesis and stabilization, remain to be elucidated. Moreover, the underlying mechanisms, safety, and feasibility of targeting TSP-4 as a therapeutic approach for OA-related pain require further investigation.

## 5. Conclusion and future perspectives

In conclusion, the glycoprotein family TSPs emerge as key regulators in the pathogenesis of OA. The presence of TSPs in articular cartilage facilitates the synthesis and assemble of ECM, promotes the chondrogenesis and chondrocyte proliferation, preserves the structural integrity of cartilage. Whereas in the synovium and subchondral bone, TSPs may participate in inflammatory response, cell differentiation, angiogenesis, and excitatory synaptogenesis through various signaling pathways. The targeting of TSPs may hold significant potential in the delay of cartilage degeneration, promotion of cartilage regeneration, attenuation of synovial inflammation, inhibition of subchondral bone remodeling, and relieving of pain, all crucial aspects for the treatment of OA. Despite the promise, challenges remain. Further investigation is still necessary to elucidate the precise molecular mechanisms through which TSPs contribute to the development and progression of OA. These findings may encourage the development of molecules, antibodies, or other biological agents targeting specific TSP functions in OA treatment. Moreover, large-scale clinical studies are imperative to validate the sensitivity and accuracy of TSPs for diagnosing OA, as well as establish standardized detection methods for clinical application.

## Figures and Tables

**Table 1:** Overview of skeletal and articular cartilage phenotypes in Thrombospondins knockout mice

Knockout gene	Skeleton phenotype	Articular cartilage phenotype	Ref.
TSP-1	Increased cancellous and cortical bone mass Mild lordotic curvature of the spine; craniofacial dysmorphism	growth plate disorganization	19; 20
TSP-2	Increased endosteal bone thickness in adult mice; exhibited a brittle phenotype on cortical bone associated with changes in collagen fibrillogenesis. Accelerated vascularization and bone formation at the fracture site	Reduced Col2a and Sox9 expression, reduced cartilage formation at the fracture site.	21; 22; 23
TSP-3	Acceleration in the rate of femoral head endochondral ossification	Increased hypertrophy in growth plate chondrocytes; growth plate disorganization	24; 25
TSP-4	No phenotypic differences were observed	Transient thinning of articular cartilage, but no significant effects on cell proliferation, metabolism and apoptosis	26
TSP-5	Pseudoachondroplasia; multiple epiphyseal dysplasia; joint abnormalities and short stature	Growth plate disorganization; Decreasing in the number of growth plate chondrocytes; early onset of OA	25; 27

**Table 2:** Summarizing the role of thrombospondins in inflammatory response related to OA and RA.

Gene	Disease	Cell source	Expression pattern	Intervention	*In-vivo* model	Outcome	Ref.
TSP-1	OA	-	Decreased TSP-1 in synovium and synovial fluid from OA rats	Intraarticular injection of TSP-1 adenovirus.	ACLT induced OA rats	Reduced angiogenesis, inflammation, macrophage infiltration, and cartilage degradation; Inhibited T lymphocyte proliferation.	41; 59
	RA	FLSs from RA patients	Increased TSP-1 in synovium and plasma from RA patients	Intraperitoneal injection of TSP1-derived peptide	Peptidoglycan- polysaccharide induced erosive arthritis in rats	TSP1-derived peptide reduced inflammation, angiogenesis and pannus formation; TNF-α inhibitors restored TSP-1 levels and reduce inflammation.	50; 51; 60
	Obesity related inflammation	BMDMs and human macrophages	Increased TSP-1 in developing adipose tissue from obese mice and humans.	Treatment with recombinant TSP-1	Wild-type and TLR4-deficient mice	TSP-1 activated TLR4 signaling in macrophages and induced TNF-α production	57
TSP-2	OA	FLSs from OA patients	Increased TSP-2 expression compared to normal synovial fibroblasts.	TSP2-neutralizing antibody	ACLT induced OA rats	TSP2 activated the PI3K/Akt/NF-κB pathway through integrin αvβ3, inducing IL-6 and inflammation, while its neutralizing antibodies reduced cartilage damage and inflammation.	52
	RA	FLSs from RA patients	Decreased TSP-2 expression in RA synovium.	Overexpression of LINC01197 (which sponges miR-150 to promote TSP-2 expression); miR-150 mimic; TSP-2 overexpression	Collagen-induced arthritis RA mice	TSP-2 overexpression inhibited RA-FLS proliferation and inflammatory responses; TSP-2 expression was increased by LINC01197, leading to reduced RA severity, swelling, and inflammation in RA mice.	61
TSP-4	Acute inflammation and peritonitis	BMDMs and RAW264.7 cells	Increased TSP-4 expression under the stimulation of LPS, GM-CSF and IFN-γ.	TSP-4 knockout mice	C57BL/6 mice with LPS-induced peritonitis	TSP-4 promoted pro-inflammatory macrophages polarization	54

**Table 3:** Application of thrombospondins in cartilage regeneration

Gene	Cell source	Modification	*In-vivo* model	Outcome	Ref.
TSP-1	ADSCs	Lentivirus transfection	Subcutaneously transplanted the cell-scaffolds into nude mice for 8 weeks.	Reduced angiogenesis and osteogenic differentiation *in vitro*; enhanced chondrogenic differentiation and inhibited osteogenic differentiation *in vivo*.	127
	BMSCs and ADSCs	Recombinant TSP-1 and siRNA transfection	Intraarticular injection of TSP-1-siRNA transfected ADSCs in CIOA mouse model.	Enhanced chondrogenic differentiation of BM-MSCs *in vitro*; impaired chondroprotective after TSP-1 knockdown *in vivo*	59
	BMSCs	Combination of recombinant TSP-1 and recombinant osteogenic protein-1 (OP-1)	TSP-1 and OP-1 containing granules pressed into the microfracture holes in femoral trochlea of miniature pigs.	Inhibited angiogenesis *in vitro*; inhibited chondrocyte hypertrophy, endochondral ossification and angiogenesis *in vivo*.	137
	Chondrosarcoma cell line HCS-2/8	Recombinant TSP-1 incorporated gelatin hydrogel	Implantation of rTSP-1 incorporated hydrogel into the microfracture holes in femoral trochlea of rat; Intraarticular injection of rTSP-1 incorporated hydrogel in MIA rat.	Enhanced proteoglycan synthesis *in vitro*; enhanced cartilage regeneration in both surgically and chemically induced OA model.	138
TSP-2	BMSCs, hUCB-MSCs and chondroprogenitor cells	Treatment with synovial fluid from OA patients, recombinant TSP-2 and TSP-2-siRNA	Transplantation of rTSP-2 along with hyaluronic acid gel composite into the defect area in femoral trochlea of New Zealand white rabbits	Increased chondrogenic differentiation and inhibited hypertrophic differentiation, decreased ECM synthesis after TSP-2 knockdown *in vitro*; increased cartilage regeneration *in vivo*.	129; 130; 131
	ADSCs	Treatment with recombinant TSP-2	Intraarticular injection of recombinant TSP-2 in ACLT induced OA rabbits.	Increased chondrogenic differentiation *in vitro*; Increased cartilage regeneration and decreased inflammatory cytokines secretion *in vivo*.	128
TSP-5	BMSCs and mesenchymal fibroblasts (C3H10T1/2)	Plasmid and lipid DNA transfection	-	Enhanced chondrogenic differentiation and ECM organization and assembly *in vitro*.	133; 134; 135
	BMSCs	Adenovirus transfection	Transplantation of TSP-5 transfected BMSCs loaded biphasic scaffold into the defect area in femoral trochlea of New Zealand white rabbits.	Increased glycosaminoglycans synthesis *in vitro*; enhanced osteochondral defect repair, cartilage regeneration, compressive modulus *in vivo*.	132
